# Acute and Subacute Toxicity of Sialidase From *Clostridium perfringens* Type A in Mice (*Mus musculus*): Organ-Specific Damage and Immune Response

**DOI:** 10.1155/vmi/5582663

**Published:** 2025-07-01

**Authors:** Otto Sahat Martua Silaen, Christian Marco Hadi Nugroho, Ryan Septa Kurnia, Silvia Tri Widyaningtyas, I Wayan Teguh Wibawan, R. Tedjo Sasmono, Amin Soebandrio

**Affiliations:** ^1^Doctoral Program in Biomedical Science, Faculty of Medicine, University of Indonesia, Jakarta 10430, Indonesia; ^2^Animal Health Diagnostic Unit, PT Medika Satwa Laboratoris, Bogor, Indonesia; ^3^Faculty of Medicine, Virology and Cancer Pathobiology Research Centre, University of Indonesia, Jakarta 10430, Indonesia; ^4^Division of Medical Microbiology, School of Veterinary Medicine and Biomedical Sciences, IPB University, Bogor 16680, Indonesia; ^5^Eijkman Research Center for Molecular Biology, National Research and Innovation Agency, Cibinong 16911, Indonesia; ^6^Department of Microbiology, Faculty of Medicine, University of Indonesia, Jakarta 10320, Indonesia

**Keywords:** antiviral, *Clostridium perfringens*, sialic acid, sialidase, toxicity

## Abstract

Sialidases, enzymes produced by *Clostridium perfringens* Type A, play a critical role in cleaving sialic acid residues essential for viral entry into host cells. By targeting pathogens such as coronaviruses, influenza, and paramyxoviruses, sialidase represents a promising therapeutic candidate. While in vitro studies confirm its efficacy against influenza, evaluating its safety profile in vivo is imperative. This study investigates the acute and subacute toxicity of sialidase from *C. perfringens* Type A in BALB/c mice (*Mus musculus*). Acute toxicity involved a single intranasal dose followed by a 14-day observation, while subacute toxicity encompassed daily doses for 30 days. Mice were administered 187.5, 375, or 750 mU/mL of sialidase, with saline as the control. No mortality or overt toxicity occurred, but significant histopathological alterations were evident in the lungs and liver at higher doses. Observed effects included lung inflammation and edema, liver congestion, and kidney inflammation. Hematological analysis revealed immunosuppressive effects, including reduced white blood cell and lymphocyte counts, alongside dose-dependent IL-6 expression changes. Sialidase doses of 187.5 and 375 mU/mL were deemed safe, whereas toxicity became pronounced at 750 mU/mL.

## 1. Introduction

Sialidases, or neuraminidases, are enzymes that catalyze the cleavage of sialic acid residues from glycoproteins and glycolipids, playing a pivotal role in diverse biological functions, including microbial pathogenesis and immune system evasion [1]. These enzymes are widely produced by microorganisms, such as bacteria, viruses, and fungi [2]. Among bacterial producers, *Clostridium perfringens*, a Gram-positive, spore-forming anaerobe, stands out for synthesizing three distinct sialidases: NanH, NanI, and NanJ [3]. These enzymes contribute to the pathogenesis of severe conditions like necrotic enteritis in poultry and gas gangrene in humans, emphasizing their role in disease progression [4].

Beyond their pathogenic implications, sialidases exhibit potential as therapeutic agents, particularly in antiviral strategies. By hydrolyzing sialic acid residues on host cell surfaces, these enzymes could disrupt viral entry mechanisms, providing a novel approach to combat viruses such as coronaviruses, influenza, paramyxoviruses, and reoviruses, all of which exploit sialic acid for cellular invasion [5–7]. Prior studies underscore the efficacy of sialidase in reducing viral replication; for instance, a 375 mU/mL sialidase dose effectively inhibited paramyxovirus replication in chicken embryo fibroblasts by modulating toll-like receptor (TLR) and interferon (IFN) responses [8–11]. However, despite promising in vitro data, the transition of sialidase to clinical application necessitates rigorous in vivo evaluation to establish its safety profile and therapeutic viability.

The current study focuses on the acute and subacute toxicity assessment of sialidase derived from *C. perfringens* Type A, administered intranasally in BALB/c mice (*Mus musculus*). This route reflects potential therapeutic applications, targeting respiratory pathogens reliant on sialic acid for infection. Toxicity evaluations span clinical observations, hematological and biochemical profiling, and histopathological examination of major organs, alongside IL-6 gene expression analysis to elucidate immune responses and inflammatory pathways. Establishing a safe and effective dosing regimen for sialidase is imperative to advancing its development as an antiviral agent, particularly against sialic acid–utilizing viruses.

## 2. Materials and Methods

### 2.1. Bacterial Culture and Sialidase Purification

The *C. perfringens* Type A bacteria used in this study, carrying the NanH, NanI, and NanJ sialidase genes, were archived isolates from PT. Medika Satwa Laboratoris. In our prior work [3], we produced the native sialidase under anaerobic conditions at 37°C overnight, while keeping the pH controlled at 7. The *C. perfringens* propagation medium comprised trypticase, yeast extract, cysteine hydrochloride, and NaCl at a concentration of 1.0% and a pH of 7.4. Finally, the culture was chilled and subjected to centrifugation to eliminate cells. In order to deactivate toxin activity, the isolated supernatant was acidified to a pH of 5, and the protein was precipitated using ammonium sulfate. The dialyzed residue underwent additional purification by ion exchange chromatography with Q Sepharose® Fast Flow (Merck, Germany), followed by affinity chromatography with oxamic acid agarose [12]. It was then kept at a temperature of −20°C. The pure sialidase enzyme activity was evaluated using the Neuraminidase Assay Kit MAK121 (Sigma-Aldrich) following the manufacturer's instructions to determine a quantitative value in units per milliliter (U/mL).

### 2.2. Animals

PT. Bio Farma (Bandung, Indonesia) provided 40 healthy adult male BALB/c mice (*Mus musculus*) aged 6 weeks and weighing between 20 and 30 g. Within the Experimental Animal Laboratory of PT. Medika Satwa Laboratoris, the animals were accommodated in polypropylene cages measuring 30 × 19 × 13 cm. These cages were equipped with stainless-steel wire covers and a bed of pine shavings. Each cage could accommodate a maximum of five animals. The mice were maintained at ambient temperature (25 ± 2°C) with a 12 h artificial light/dark cycle. Food from PT. Citra Ina Feedmill in Jakarta, Indonesia, was supplied and water *ad libitum*. Animals were acclimated to laboratory conditions for 15 days prior to experimentations [13].

### 2.3. Ethics Declaration

The animal experiments were carried out in strict compliance with the National Institutes Guide for the Care and Use of Laboratory Animals. The Animal Use Ethics Committee of the Faculty of Medicine, Universitas Indonesia, approved the operations under protocol number KET-833/UN2.F1/ETIK/PPM.00.02/2024.

### 2.4. Acute Toxicity Assessment

Acute toxicity was conducted following the protocol outlined in Guideline 423 of the Organisation for Economic Co-operation and Development (OECD) [14]. Animals were randomly divided into four groups (*n* = 5). The control group received a single intranasal dose of phosphate-buffered saline (PBS) at pH 7.3. The low-dose group received a single intranasal administration of sialidase at 187.5 mU/mL, while the middle-dose group received 375 mU/mL, and the high-dose group received 750 mU/mL. Body weight was monitored daily throughout 14 days. The percentage change in body weight was calculated as % change = ((body weight at time *t* − initial body weight)/initial body weight) × 100. On the final day of observation, all mice were euthanized by intraperitoneal injection of 9 mg/kg ketamine (10%) and 10 mg/kg xylazine (2%).

### 2.5. Subacute Toxicity Assessment

The subacute toxicity assessment for repeated doses of sialidase followed the methodology described in OECD Guideline 407 [15]. The mice were randomly assigned to four groups (*n* = 5). The control group received a single intranasal dose of PBS, pH 7.3, daily for 30 days. The low-dose group received a daily intranasal dose of sialidase at 187.5 mU/mL. The middle-dose group received sialidase at 375 mU/mL, and the high-dose group received 750 mU/mL, administered intranasally for 30 days. Daily observations were made for any signs of mortality or abnormal clinical symptoms, and body weight was recorded. The percentage change in body weight was calculated as % change = ((body weight at time *t* − initial body weight)/initial body weight) × 100. On the final day, animals were euthanized using the same method as in the acute toxicity test, and blood was collected via cardiac puncture to obtain the total available volume.

### 2.6. Histopathological Parameters

Following euthanasia, 1 cm-thick tissue samples from the nasal cavity, lungs, liver, and kidneys were collected from both the acute and subacute toxicity tests for histological analysis. Each sample was sectioned sagittally and preserved in 10% buffered formalin for 24 h. For histopathological examination, the tissues were processed by gradual dehydration with increasing concentrations of ethanol (70°–100°), cleared in xylene, embedded in paraffin, and sectioned with a microtome to produce 3.0 μm-thick slices. These sections were then stained with hematoxylin and eosin (HE) and examined microscopically at 40× and 100× magnifications. A qualified histopathologist independently assessed each slide, and a second certified histopathologist reviewed the findings for verification [16].

The lung histopathology scoring system used in this study was adapted from Hulse et al. [17], with modifications emphasizing airway inflammation through six indicators of lung injury. Each indicator was assessed semiquantitatively on a scale of 0 to 3, producing a possible total score of 18 points. The liver histopathology scoring system was modified from Krishna et al. [18] targeting four indicators of liver injury. Each parameter was scored from 0 to 3, yielding a maximum score of 12 points. For kidney histopathology, a scoring system was adapted from Chang et al. [19] focusing on four indicators of renal injury, each scored from 0 to 3, for a total score of up to 12 points.

### 2.7. Hematological and Biochemical Parameters

At the end of the subacute toxicity study, mice were sedated with ketamine (9 mg/kg) and xylazine (10 mg/kg) before blood collection via cardiac puncture. Whole blood in EDTA tubes was analyzed for hematological parameters, including white blood cell (WBC), LYM, RBC, HGB, HCT, MCV, MCH, mean corpuscular hemoglobin concentration (MCHC), and PLT, using an ONETECH Full Automated Hematology Analyzer (Yueshen Medical Equipment, China). Plasma was separated by centrifugation and analyzed for AST and ALT as markers of liver function, and BUN as a kidney function marker, using an automated biochemical system with commercial reagents (Reid Diagnostic, Italy) at 37°C. Measurements were performed using a Genesys 10s UV/VIS spectrophotometer (Thermo Fisher Scientific, USA) [20].

### 2.8. IL-6 Gene Expression Parameter

IL-6 gene expression was quantified via RT-qPCR using GAPDH as the normalization control. Liver, lung, and spleen tissues were collected posteuthanasia, and RNA was extracted using the ReliaPrep RNA Miniprep System (Promega, WI, USA). cDNA synthesis was performed with the ReverTraAce cDNA Synthesis Kit (Toyobo, Osaka, Japan) using 1 μg RNA. Quantitative RT-qPCR was conducted with SensiFAST™ SYBR® Lo-Rox Kit (Bioline, TN, USA). Primer sequences were IL-6 Forward (5′-CCAGAAACCGCTATGAAGTTCC-3′), IL-6 Reverse (5′-TTGTCACCAGCATCAGTCCC-3′) [19], GAPDH Forward (5′-CATGGCCTTCCGTGTTCCTA-3′), and GAPDH Reverse (5′-ACTTGGCAGGTTTCTCCAGG-3′) [21]. The specificity of amplification was confirmed via melting curve analysis, and relative expression (RQ) was calculated using the 2^−ΔΔCt^ method.

### 2.9. Statistical Analysis

The quantitative data were analyzed using the statistical software SPSS and GraphPad Prism 9.1.2. Presented as means ± standard error of the mean (SEM) or standard deviation (SD), all data represent multiple experiments. Differences were deemed statistically significant when the *p* value was less than 0.05, computed using analysis of variance (ANOVA).

## 3. Results

### 3.1. Acute Toxicity Assessment

Sialidase, when administered intranasally at doses of 187.5, 375, and 750 mU/mL in mice, did not induce mortality or visible toxic symptoms. All treated mice exhibited normal behavior and survived the 14 day experimental period. However, body weight changes varied among groups, and histopathological alterations were noted in the lungs, liver, and kidneys.

**Figure 1:**
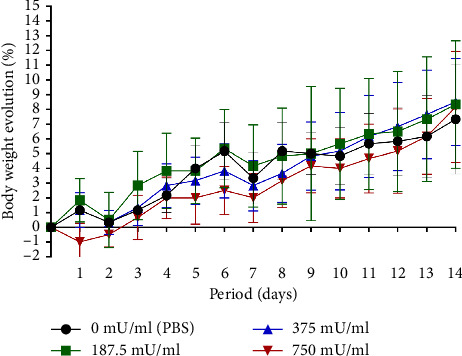
Changes in body weight (%) of *Mus musculus* over 14 days following intranasal administration of sialidase at different doses.

The 750 mU/mL group exhibited lower body weight changes compared to the other groups. However, during the final 2 days of observation, an upward trend in weight change was noted, resulting in an overall percentage increase comparable to that of the 187.5 and 375 mU/mL groups. A distinct pattern was observed in the 750 mU/mL group, with an initial weight reduction on the first posttreatment day, whereas other groups exhibited a similar decline on the second day posttreatment (Figure 1).

### 3.2. Histopathological Analysis in Acute Toxicity

**Figure 2:**
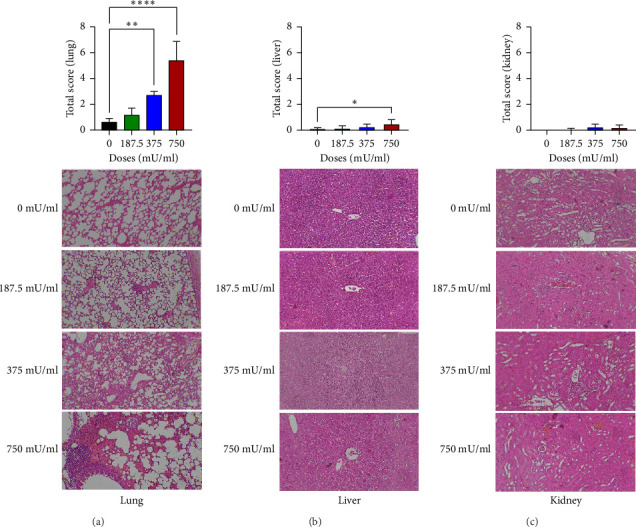
Total histopathological damage scores for lung (a), liver (b), and kidney (c) tissues following intranasal sialidase administration. Data are presented as mean ± SEM. An asterisk (∗) denotes statistically significant differences (*p* < 0.05). Representative histological images of lung, liver, and kidney tissues are shown below the corresponding graphs. All histological images were captured at the same magnification (40×) to ensure consistency in tissue comparisons.

Histopathological examination revealed dose-dependent alterations in lung and liver tissues, whereas kidney morphology remained largely unaffected. Lung damage was significantly increased in the 375 and 750 mU/mL sialidase-treated groups (*p* < 0.05) compared to controls, indicating pulmonary toxicity at higher doses (Figure 2(a)). Liver histopathology showed significant tissue damage (*p* < 0.05) at 750 mU/mL but not at lower doses (*p* > 0.05) (Figure 2(b)). In contrast, kidney sections exhibited no significant damage across all tested doses (*p* > 0.05) (Figure 2(c)).

### 3.3. Subacute Toxicity Assessment

**Figure 3:**
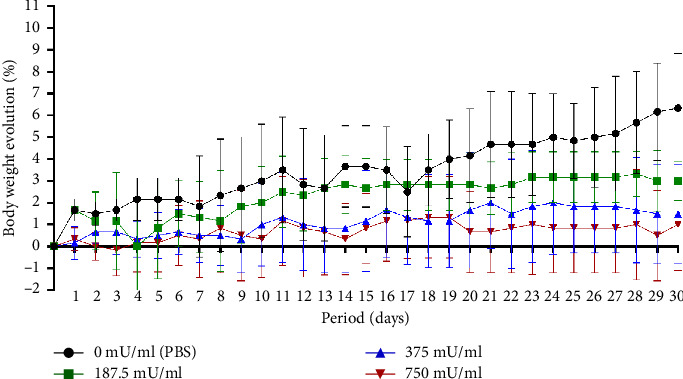
Changes in body weight (%) of *Mus musculus* over 30 days following intranasal administration of sialidase at different doses.

The body weight analysis over the 30-day subacute toxicity period showed that the 750 mU/mL group had the lowest overall body weight changes. The 187.5 mU/mL and 375 mU/mL groups exhibited greater daily body weight variations, while the control group (0 mU/mL, PBS) demonstrated the highest body weight change by day 30. No mortality was observed in any treatment group throughout the study (Figure 3).

### 3.4. Histopathological Evaluation of Lung, Liver, and Kidney Damage in Subacute Toxicity Assessment

The histopathological evaluation of lung damage during the subacute toxicity assessment is shown in Table 1. There were no significant differences (*p* > 0.05) among the treatment groups regarding the presence of neutrophils in the bronchial lumen, bronchial/bronchiolar necrosis, or hemorrhage. However, significant increases (*p* < 0.05) were observed in perivascular inflammation/fibrin and alveolar edema only in the 750 mU/mL dose group. Unlike the other indicators, significant increases (*p* < 0.05) in alveolar inflammation were noted in the 750 mU/mL dose group compared to the 187.5 mU/mL dose group.

The assessment of liver damage during subacute toxicity testing is shown in Table 2. Regarding congestion, a significant increase (*p* < 0.05) was observed in the 375 mU/mL and 750 mU/mL dose groups. In contrast, the 187.5 mU/mL dose group showed a tendency toward decreased congestion, although the difference was not significant (*p* > 0.05). Significant increases (*p* < 0.05) in portal inflammation were observed only in the 750 mU/mL dose group. Unlike the previous indicators, no significant changes (*p* > 0.05) were noted in interface hepatitis across all treatment groups. Significant results (*p* < 0.05) were observed in all treatment groups for lobular inflammation.

The observations and assessment of kidney, liver, and lung damage in mice during subacute toxicity testing are tabulated based on the respective organ evaluations. The assessment of kidney damage is shown in Table 3. Congestion in the kidneys increased with the dose of sialidase, with the control group (0 mU/mL) showing low congestion. Significant increases (*p* < 0.05) in congestion were observed at 375 mU/mL and 750 mU/mL doses compared to the control and low-dose (187.5 mU/mL) groups. Hemorrhagic indicators showed significant differences (*p* < 0.05) only in the highest dose group (750 mU/mL). Unlike the previous indicators, there were no significant differences (*p* > 0.05) in necrosis across all treatment groups, although necrosis tended to increase with the dose. No inflammation was detected in the control group and the 187.5 mU/mL dose group, while significant increases (*p* < 0.05) in inflammation were observed at 375 mU/mL and more prominently at 750 mU/mL doses.

**Figure 4:**
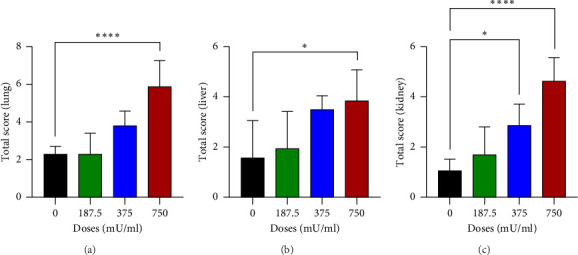
Analysis of total damage scores for all indicators in organs: (a) lungs, (b) liver, and (c) kidneys.

The analysis of total damage scores across all indicators in each organ revealed distinct patterns of toxicity. A significant increase (*p* < 0.05) in lung damage was observed at the highest dose (750 mU/mL), as shown in Figure 4(a). Similarly, the liver damage score significantly increased (*p* < 0.05) in the 750 mU/mL dose group (Figure 4(b)). In contrast, kidney damage exhibited a dose-dependent increase, with significant changes (*p* < 0.05) detected at both 375 and 750 mU/mL doses (Figure 4(c)). These findings collectively indicate that while all three organs were affected, the severity and dose–response relationship varied, with the kidneys showing earlier signs of toxicity compared to the lungs and liver.

### 3.5. Hematological and Blood Chemistry Analysis in Subacute Toxicity Assessment

**Figure 5:**
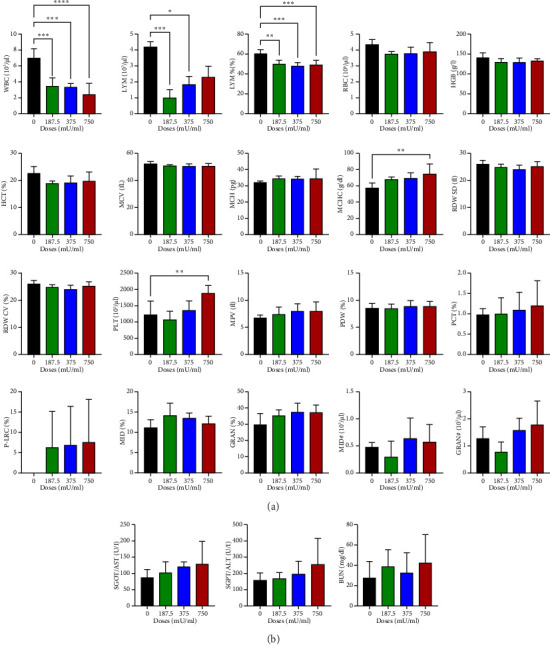
Diagrams of general hematological values (a) and blood chemistry (b) in mice (*Mus musculus*).

General hematological examinations were also conducted to assess hemoglobin, hematocrit, erythrocytes, leukocytes, platelets, and other blood components after subacute toxicity testing (Figure 5(a)). The results showed that all sialidase dose groups experienced a significant reduction (*p* < 0.05) in WBC count. Sialidase appeared to cause a significant decrease (*p* < 0.05) in lymphocyte count in mice, particularly at doses of 187.5 mU/mL and 375 mU/mL. At the 750 mU/mL dose, lymphocyte counts began to increase (*p* > 0.05) but remained below control levels. The analysis of lymphocyte percentages in sialidase-treated mice indicated a significant reduction (*p* < 0.05) at all sialidase dose levels. Another parameter that changed was the MCHC, with a significant increase (*p* < 0.05) only observed in the 750 mU/mL dose group. A significant increase (*p* < 0.05) was also noted in the same dose group for platelet count.

Although some parameters differed from the control, no significant differences (*p* > 0.05) were observed in RBC, HGB, HCT, MCV, MCH, RDW, MPV, PDW, PCT, P-LRC, MID%, MID#, GRAN%, and GRAN# values (Figure 5(a)). The biochemical analyses of blood, including AST, ALT, and BUN, also did not show significant differences (*p* > 0.05) between the treatment groups. However, the 750 mU/mL dose group exhibited an overall increase in these blood chemistry levels (Figure 5(b)).

### 3.6. IL-6 Gene Expression

**Figure 6:**
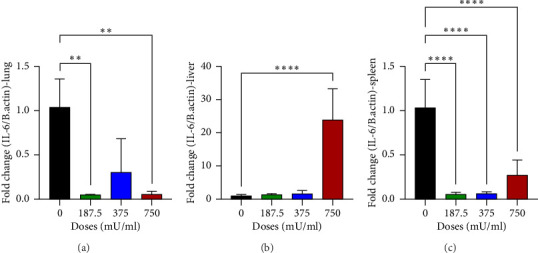
Relative expression of IL-6 gene in the lungs (a), liver (b), and spleen (c) of mice (*Mus musculus*).

The analysis of IL-6 gene expression yielded varied results as shown in Figure 6. In the lungs, a significant decrease (*p* < 0.05) was observed in the 187.5 mU/mL and 750 mU/mL dose groups (Figure 6(a)). In contrast, a significant increase (*p* < 0.05) in IL-6 expression was noted in the liver at the 750 mU/mL dose (Figure 6(b)). A different pattern was observed in the spleen, where IL-6 gene expression showed a significant decrease (*p* < 0.05) across all sialidase treatment groups compared to the control group (Figure 6(c)).

## 4. Discussion

This study evaluated the acute and subchronic toxicity of sialidase from *Clostridium perfringens* Type A in BALB/c mice (*Mus musculus*), focusing on its safety profile. By cleaving sialic acid residues, sialidase plays a dual role in microbial pathogenicity and immune modulation, making it a potential antiviral agent against pathogens like avian influenza H5N1 that depend on sialic acid for host cell entry.

Intranasal administration of sialidase at doses up to 750 mU/mL did not result in mortality or observable toxic symptoms, affirming a favorable acute toxicity profile. These results are consistent with prior studies on MDCK cells and in ovo embryo models, which reported minimal acute toxicity [3, 22]. However, the transient reduction in body weight observed on the first day posttreatment at 750 mU/mL suggests a potential mild physiological response or stress reaction. This may involve transient metabolic shifts, immune activation, or mild inflammation triggered by the administration route or dosage. Further investigation into stress markers, such as cortisol levels or early inflammatory cytokines, could elucidate the mechanisms underlying this response.

Histopathological analyses revealed dose-dependent organ damage, with significant effects observed in the lungs and liver at 375 mU/mL and 750 mU/mL. In the lungs, perivascular inflammation, alveolar edema, and alveolar inflammation were prominent, indicating localized pulmonary inflammatory responses at higher doses. Sialidase activity is linked to pulmonary fibrosis, as evidenced by its upregulation in fibrotic lesions and involvement in inflammatory pathways. Previous study aligns with our findings of alveolar edema and inflammation, suggesting a role for sialidase in exacerbating inflammatory and fibrotic responses in lung tissue [23].

Similarly, liver histology demonstrated increased congestion and portal inflammation, suggesting hepatocellular stress. Sialidase-mediated desialylation influences liver stress responses by altering glycosylation patterns, which can affect hepatocyte function and immune signaling. This desialylation process has been shown to potentiate organ inflammation, which might explain the congestion and portal inflammation observed. Furthermore, the liver's central role in systemic immune modulation could amplify these effects during high-dose sialidase exposure [23, 24].

The kidneys showed dose-dependent congestion and inflammatory changes, although these effects were less severe than those observed in the lungs and liver. This differential organ susceptibility underscores the need for targeted monitoring of pulmonary and hepatic functions during sialidase exposure.

In subacute toxicity assessment, mice receiving the highest dose of sialidase (750 mU/mL) exhibited the lowest body weight change over the 30-day period, which might reflect a cumulative toxic effect over time. This is consistent with the histopathological findings, where significant damage was observed in the lungs, liver, and kidneys at higher doses [25, 26]. Notably, the presence of significant perivascular inflammation, alveolar edema, and alveolar inflammation in the lungs at 750 mU/mL underscores the potential for sialidase to induce chronic inflammatory responses in pulmonary tissues. Similarly, the significant increase in liver congestion and portal inflammation at the highest dose indicates that prolonged exposure to sialidase could lead to liver injury. The kidney showed dose-dependent increases in congestion and inflammation, further supporting the conclusion that repeated sialidase exposure can lead to progressive organ damage.

The hematological analysis revealed a significant reduction in WBC counts across all sialidase-treated groups, indicating a possible immunosuppressive effect. The decrease in lymphocyte counts, particularly at lower doses, suggests that sialidase may impair lymphocyte proliferation or survival [26]. However, the observed increase in lymphocyte counts at 750 mU/mL, although not significant, may reflect a compensatory response or a shift in immune dynamics over time. The significant increase in MCHC and platelet counts at the highest dose could be indicative of altered erythropoiesis and thrombopoiesis, possibly due to the inflammatory response induced by sialidase. Biochemical markers, including AST, ALT, and BUN, displayed trends toward increased levels at 750 mU/mL, indicative of mild hepatic and renal stress. Although not statistically significant, these findings warrant attention, as prolonged exposure or higher doses could exacerbate organ stress.

Analysis of IL-6 gene expression offered insights into the inflammatory responses elicited by sialidase. A significant reduction in IL-6 expression in the lungs at 187.5 and 750 mU/mL indicates potential suppression of local inflammatory signaling, possibly due to immunomodulatory effects. This aligns with previous studies suggesting that certain bacterial sialidases can modulate immune responses by altering sialylation patterns on host cells [27, 28]. Conversely, the liver showed significantly increased IL-6 expression at 750 mU/mL, consistent with histopathological evidence of liver inflammation. Elevated IL-6 levels in the liver may be indicative of hepatocellular injury and activation of proinflammatory pathways, as described in earlier research on cytokine-mediated liver damage [29, 30]. The spleen exhibited consistently downregulated IL-6 expression across all doses, suggesting systemic immunosuppressive effects that may contribute to the observed hematological changes.

This study underscores the dose-dependent toxicological effects of sialidase, highlighting its relative safety at lower doses while identifying risks associated with higher concentrations. The organ-specific damage, particularly in the lungs and liver, necessitates careful dose optimization for future therapeutic applications. Additionally, changes in hematological and inflammatory parameters emphasize the need for comprehensive evaluations of sialidase's broader immunological impacts.

Future research should focus on the long-term effects of sialidase exposure, its pharmacokinetics, and biodistribution to establish a robust safety and efficacy profile. Investigating stress pathways and markers such as heat shock proteins or cytokine profiles could provide further insights into transient physiological responses. Furthermore, given the observed IL-6 modulations, future studies could explore the therapeutic potential of sialidase in inflammatory diseases where cytokine modulation is critical.

## 5. Conclusions

In conclusion, sialidase from *Clostridium perfringens* Type A is generally well tolerated at lower dose. The observed changes in hematological parameters and IL-6 gene expression further underscore the potential for sialidase to modulate immune responses, which could have implications for its therapeutic use. These results highlight the importance of dose optimization and monitoring for potential adverse effects in the development of sialidase as an antiviral agent. Future studies should focus on elucidating the mechanisms underlying sialidase-induced toxicity and exploring strategies to mitigate these effects while harnessing the enzyme's therapeutic potential.

## Figures and Tables

**Table 1:** Histopathological assessment of lung damage during subacute toxicity testing.

Dose groups (mUmL)	Parameters					
	Neutrophils in the bronchial lumen	Bronchial/bronchiolar necrosis	Perivascular inflammation/fibrin	Alveolar edema	Alveolar inflammation	Hemorrhage
0	0.48 ± 0.41^a^	0.36 ± 0.33^a^	0.44 ± 0.17^a^	0.60 ± 0.51^a^	0.20 ± 0.24^ab^	0.24 ± 0.17^a^
187.5	0.32 ± 0.30^a^	0.32 ± 0.11^a^	0.56 ± 0.30^a^	0.80 ± 0.71^a^	0.08 ± 0.18^a^	0.28 ± 0.30^a^
375	0.80 ± 0.24^a^	0.48 ± 0.46^a^	0.72 ± 0.30^a^	0.68 ± 0.30^a^	0.80 ± 0.45^ab^	0.36 ± 0.36^a^
750	0.60 ± 0.45^a^	0.72 ± 0.36^a^	1.24 ± 0.62^b^	1.52 ± 0.36^b^	1.36 ± 0.65^b^	0.52 ± 0.30^a^

*Note:* Different superscripts (a, b, c, d) indicate significant differences within a column (*p* < 0.05).

**Table 2:** Histopathological assessment of liver damage during subacute toxicity testing.

Dose groups (mU/mL)	Parameters			
	Congestion	Portal inflammation	Interface hepatitis	Lobular inflammation
0	0.64 ± 0.48^a^	0.40 ± 0.37^a^	0.00 ± 0.00^a^	0.08 ± 0.18^a^
187.5	0.56 ± 0.46^ab^	0.92 ± 0.63^ab^	0.16 ± 0.17^a^	0.32 ± 0.39^b^
375	1.00 ± 0.49^bc^	1.36 ± 0.43^ab^	0.32 ± 0.36^a^	0.84 ± 0.17^c^
750	1.20 ± 0.51^c^	1.56 ± 0.64^b^	0.44 ± 0.36^a^	1.60 ± 0.27^d^

*Note:* Different superscripts (a, b, c, d) indicate significant differences within a column (*p* < 0.05).

**Table 3:** Histopathological assessment of kidney damage during subacute toxicity testing.

Dose groups (mU/mL)	Parameters			
	Congestion	Hemorrhagic	Necrosis	Inflammation cells
0	0.56 ± 0.30^a^	0.40 ± 0.14^a^	0.12 ± 0.18^a^	0.00 ± 0.00^a^
187.5	0.76 ± 0.43^a^	0.72 ± 0.36^a^	0.24 ± 0.43^a^	0.00 ± 0.00^a^
375	1.40 ± 0.51^b^	0.68 ± 0.33^a^	0.52 ± 0.33^a^	0.28 ± 0.63^ab^
750	1.68 ± 0.41^b^	1.44 ± 0.38^b^	0.76 ± 0.26^a^	2.80 ± 0.62^b^

*Note:* Different superscripts (a, b, c, d) indicate significant differences within a column (*p* < 0.05).

## Data Availability

Research data are not shared.

## References

[1] Juge N., Tailford L., Owen C. D. (2016). Sialidases From Gut Bacteria: A Mini-Review. *Biochemical Society Transactions*.

[2] Belser J. A., Lu X., Szretter K. J. (2007). DAS181, A Novel Sialidase Fusion Protein, Protects Mice From Lethal Avian Influenza H5N1 Virus Infection. *The Journal of Infectious Diseases*.

[3] Kurnia R. S., Tarigan S., Nugroho C. M. H. (2022). Potency of Bacterial Sialidase *Clostridium perfringens* as Antiviral of Newcastle Disease Infections Using Embryonated Chicken Egg in Ovo Model. *Veterinary World*.

[4] Li J., Uzal F. A., McClane B. A. (2016). *Clostridium perfringens* Sialidases: Potential Contributors to Intestinal Pathogenesis and Therapeutic Targets. *Toxins*.

[5] Hedlund M., Aschenbrenner L. M., Jensen K., Larson J. L., Fang F. (2010). Sialidase‐Based Anti-Influenza Virus Therapy Protects Against Secondary Pneumococcal Infection. *The Journal of Infectious Diseases*.

[6] Zenilman J. M., Fuchs E. J., Hendrix C. W. (2015). Phase 1 Clinical Trials of DAS181, an Inhaled Sialidase, in Healthy Adults. *Antiviral Research*.

[7] Malakhov M. P., Aschenbrenner L. M., Smee D. F. (2006). Sialidase Fusion Protein as a Novel Broad-Spectrum Inhibitor of Influenza Virus Infection. *Antimicrobial Agents and Chemotherapy*.

[8] Worrall E. E., Sudarisman P. A., Priadi A. (2009). Sialivac: An Intranasal Homologous Inactivated Split Virus Vaccine Containing Bacterial Sialidase for the Control of Avian Influenza in Poultry. *Vaccine*.

[9] Nugroho C. M. H., Kurnia R. S., Tarigan S. (2022). Screening and Purification of NanB Sialidase From *Pasteurella multocida* With Activity in Hydrolyzing Sialic Acid Neu5Acα(2–6)Gal and Neu5Acα(2-3)Gal. *Scientific Reports*.

[10] Matrosovich M., Herrler G., Klenk H. D. (2015). Sialic Acid Receptors of Viruses. *Topics in Current Chemistry*.

[11] Kurnia R. S., Setiawaty R., Natih K. K. N. (2022). Evaluation of Inhibitor Activity of Bacterial Sialidase From *Clostridium perfringens* Against Newcastle Disease Virus in the Cell Culture Model Using Chicken Embryo Fibroblast. *Journal of Advanced Veterinary and Animal Research*.

[12] Tarigan S., Indryani R., Darminto, Ignjatovic J. (2013). Purification of Neuraminidase From Influenza Virus Subtype H5N1. *Jurnal Ilmu Ternak dan Veteriner*.

[13] Turner P. V., Brabb T., Pekow C., Vasbinder M. A. (2011). Administration of Substances to Laboratory Animals: Routes of Administration and Factors to Consider. *Journal of the American Association for Laboratory Animal Science*.

[14] Angelo A. F. B., Augustin A. K., Parfait K. B. G., Nene B. S. A., Aka Francis Beranger Angelo C. (2016). Acute Toxicity of the Aqueous Extract of Roasted and Ground Beans of *Coffea canephora* Robusta in the Wistar Rat. *The Pharma Innovation Journal*.

[15] Hsu P. K., Tsai Y. T., Lin Y. C., Kuan C. M. (2022). Assessment of the Acute and Sub-Acute Toxicity of the Ethanolic Extract of the Aerial Parts of Crassocephalum Rabens (Asteraceae) in Rats. *Toxicology Reports*.

[16] Panna S. N., Nazir K. H. M. N. H., Rahman M. B. (2015). Isolation and Molecular Detection of *Pasteurella multocida* Type A From Naturally Infected Chickens, and Their Histopathological Evaluation in Artifically Infected Chickens in Bangladesh. *Journal of Advance Veterinary and Animal Research*.

[17] Hulse E. J., Smith S. H., Wallace W. A. (2020). Development of a Histopathology Scoring System for the Pulmonary Complications of Organophosphorus Insecticide Poisoning in a Pig Model. *PLoS One*.

[18] Krishna M. (2021). Histological Grading and Staging of Chronic Hepatitis. *Clinical Liver Disease*.

[19] Chang M. W., Chen C. H., Chen Y. C. (2015). Sitagliptin Protects Rat Kidneys From Acute Ischemia-Reperfusion Injury via Upregulation of GLP-1 and GLP-1 Receptors. *Acta Pharmacologica Sinica*.

[20] Sher Y. P., Hung M. C. (2013). Blood AST, ALT and UREA/BUN Level Analysis. *Bio-Protocol*.

[21] Dong Y. F., Chen Z. Z., Zhao Z. (2016). Potential Role of microRNA-7 in the Anti-Neuroinflammation Effects of Nicorandil in Astrocytes Induced by Oxygen-Glucose Deprivation. *Journal of Neuroinflammation*.

[22] Nugroho C. M. H., Silaen O. S. M., Kurnia R. S. (2025). In Vitro Antiviral Activity of NanB Bacterial Sialidase Against Avian Influenza H9N2 Virus in MDCK Cells. *Avian Pathology*.

[23] Karhadkar T. R., Chen W., Pilling D., Gomer R. H. (2022). Inhibitors of the Sialidase NEU3 as Potential Therapeutics for Fibrosis. *International Journal of Molecular Sciences*.

[24] Karhadkar T. R., Meek T. D., Gomer R. H. (2021). Inhibiting Sialidase-Induced TGF-Β1 Activation Attenuates Pulmonary Fibrosis in Mice. *Journal of Pharmacology and Experimental Therapeutics*.

[25] Gelbke H. P., Hofmann A., Owens J. W., Freyberger A. (2007). The Enhancement of the Subacute Repeat Dose Toxicity Test OECD TG 407 for the Detection of Endocrine Active Chemicals: Comparison With Toxicity Tests of Longer Duration. *Arch Toxicol.*.

[26] Balkrishna A., Sinha S., Varshney A. (2023). 28-Day Repeated Dose Toxicological Evaluation of Coronil in Sprague Dawley Rats: Behavioral, Hematological, Biochemical and Histopathological Assessments Under GLP Compliance. *Drug and Chemical Toxicology*.

[27] Yang X., Pan Y., Xu X. (2018). Sialidase Deficiency in Porphyromonas Gingivalis Increases IL-12 Secretion in Stimulated Macrophages Through Regulation of CR3, IncRNA GAS5 and miR-21. *Frontiers in Cellular and Infection Microbiology*.

[28] Lewis A. L., Lewis W. G. (2012). Host Sialoglycans and Bacterial Sialidases: A Mucosal Perspective. *Cellular Microbiology*.

[29] Wang M. J., Zhang H. L., Chen F., Guo X. J., Liu Q. G., Hou J. (2024). The Double-Edged Effects of IL-6 in Liver Regeneration, Aging, Inflammation, and Diseases. *Experimental Hematology & Oncology*.

[30] Rani R., Kumar S., Sharma A. (2018). Mechanisms of Concanavalin A-Induced Cytokine Synthesis by Hepatic Stellate Cells: Distinct Roles of Interferon Regulatory Factor-1 in Liver Injury. *Journal of Biological Chemistry*.

